# Rheology of Cement Pastes with Siliceous Fly Ash and the CSH Nano-Admixture

**DOI:** 10.3390/ma14133640

**Published:** 2021-06-29

**Authors:** Bartosz Szostak, Grzegorz Ludwik Golewski

**Affiliations:** 1 Department of Conservation of Built Heritage, Faculty of Civil Engineering and Architecture, Lublin University of Technology, Nadbystrzycka 40 Str., 20-618 Lublin, Poland; 2 Department of Structural Engineering, Faculty of Civil Engineering and Architecture, Lublin University of Technology, Nadbystrzycka 40 Str., 20-618 Lublin, Poland; g.golewski@pollub.pl

**Keywords:** concrete, CSH nano-admixture, fly ash, concrete binding acceleration

## Abstract

The use of fly ash in cement composites adversely affects its mechanical properties during the first days of mixture curing. Modern technology, in the form of an admixture containing the hydrated calcium silicates, allows to accelerate the hardening and binding process of concrete. In this paper, studies on the influence of the admixture on properties of concretes with the ordinary Portland cements (OPC) containing the addition of siliceous fly ash (FA) have been carried out. As part of the experimental research, the authors conducted a series of studies for cement pastes modified with the addition of FA and the CSH nano-admixture (NA). In order to compare the mixtures, the following tests of cement pastes were carried out: the compressive and flexural strength, heat of hydration, SEM and rheological shrinkage. The mechanical parameters were tested after 4, 8, 12 and 24 h. The hydration heat test and microstructure analysis were carried out during the first 24 h of the concrete curing. All tests were carried out on the standard samples. On the basis of the heat of hydration test, much higher hydration heat was found in mixtures modified with the NA. During the shrinkage test, a positive effect of the NA was observed—the shrinkage during the first 28 days of mixture curing was lower than in the reference samples. The application of the CSH nano-admixture to cement pastes with the addition of FA has brought positive effects. Apart from a significant increase in strength in the first 24 h of mixture curing, a reduction in the rheological shrinkage was observed. The admixture can be successfully used in the ash concretes, in which a higher early strength is required.

## 1. Introduction

Currently, intensive research on various modifications of concretes is being carried out all over the world, e.g., with the use of: nanotechnology and nanomaterials [1,2,3], wastes [4,5,6], pozzolanic additives [7,8,9] and materials with special properties [10,11,12]. Such activities are mainly aimed at creating new composites, which would be the sustainable materials [13,14]. However, the desire to create sustainable concrete requires manufacturers, technologists and scientists to conduct continuous research in the field of changing the components of concrete mixes in such a way as to obtain building materials that are as environmentally friendly as possible. One of the most common and at the same time useful mineral components used as a cement replacement is siliceous fly ash (FA).

Numerous studies have confirmed that the addition of 20% FA to the concrete mix improves many parameters of the modified composite. These include:

- mechanical properties, e.g., [15,16,17];
- fracture toughness after 28 days of curing and at later periods, e.g., [18,19,20,21];
- corrosion resistance, e.g., [22,23];
- resistance to high temperatures, e.g., [24];
- resistance to abrasion and erosion, e.g., [25];
- more effective against negative effects originated from impact and vibrations [26,27,28] as well as dynamic loads, e.g., [29,30,31,32];
- rheological parameters such as shrinkage [33] and heat of hydration;
- expansion of the alkaline-carbonate reaction [34].

The above advantages of using FA in the construction industry are undoubtedly an argument for the use of this industrial waste in the production of concrete mixtures. However, despite a number of positive effects of using FA in concrete technology, its presence as an alternative type of binder also implies some negative effects.

In numerous previous works a negative effect on the early strength, e.g., [35,36,37,38,39,40,41] and physical properties, e.g., [42,43,44,45] gain of concrete composites with FA was described. This unfavorable characteristic of this waste unfortunately limits its application in certain branches of the concrete industry [46]. Some authors [47,48,49] have presented attempts to modify FA concrete so that it can be used in precasting.

Therefore, in order to minimize and in a way eliminate this undesirable property of FA, the authors proposed a material solution consisting in the additional application to the composition of FA concrete mix, a modern nano-admixture (NA) in the form of suspension with active seeds of the C-S-H phase (CSH).

Preliminary studies presented in [50,51,52,53,54,55,56] confirmed the beneficial effect of this preparation on the mechanical parameters of concretes from the 28th day of their curing. The differences in the NA effect on the FA-modified composites and those made on pure Ordinary Portland Cement (OPC) were also compared [57,58,59,60]. In previous experiments it was also pointed out that NA with the active CSH phase seeds has a positive effect on improving the early age parameters of concretes and pastes containing FA, mainly by accelerating the initial reactions in the material microstructure. The final confirmation of this thesis could be the study of rheological parameters of composites at very early periods in connection with the analysis of the structure development in the first hours of the material curing.

In order to carry out this task, the authors tested cement pastes modified with 20% FA and the NA. The primary goal of the NA applied was to intensify the cement matrix setting and hardening processes, which were partially retarded as a result of the FA modification. During the experiments, attention was mainly paid to the important rheological parameters of the composites, i.e.,

- Heat of hydration;
- Shrinkage of the cement matrix.

Additionally, the NA effect on the strength parameters of the analyzed cement pastes during the first 24 h of their maturation was also studied. The experiments were supplemented with microstructural studies based on the evaluation of changes in the structure of the cement matrix. The Scanning Electron Microscope (SEM) [61] experiments were carried out at the same times of material curing when the hydration heat curves were inspected, and the mechanical parameters were evaluated. Thus, it was possible to relate the results of the study of rheological and strength parameters to the evaluation of morphology of the forming structures at very early periods. The studies were started as early as after 4 h of cement pastes curing.

## 2. Significance of the Study

Rheology of cementitious composites is an extremely important issue in predicting their strength, durability and usefulness in the construction industry. It is particularly important in the case of prefabricated elements, where the time of installation into the structure is sometimes counted not in days but even in hours after their formation [47,48,49]. On the other hand, besides mechanical tests, the study of hydration heat and shrinkage of the cement matrix are the basic tests to determine the influence of additives and admixtures—that is, internal factors—on the properties of the cement composite at a very early age [62,63].

Shrinkage is one of the volumetric changes in concrete which is of particular importance from a technical point of view because it is an undesirable phenomenon. This unfavorable property of cementitious composites is linked with the volume changes of cement paste [64,65]. Total concrete shrinkage consists of volume changes due to setting and hardening of the cement paste (chemical and autogenous shrinkage) and deformation due to the water loss from the concrete surface (initially plastic shrinkage and after hardening drying shrinkage) [66,67].

The phenomena affecting and having a direct relation to the shrinkage of cementitious composites was presented in [68], whereas the main factors responsible for the shrinkage of cementitious matrix in [69]. It should be noted that particular importance is attributed here to shaping the rheology of cement matrices by using additives and admixtures.

For example, in [70] it was proved that in case of mineral additives mixtures containing a 60% volume replacement of cement with slag cement exhibited reductions in shrinkage. A positive effect of the hemp fibers has also been reported [71]. Chemical admixtures are also very effective in reducing shrinkage of a cement matrix [72].

An important role is also played by admixtures in the modification of the second important rheological parameter of the concrete mix, i.e., the heat of hydration, e.g., [73,74]. Since the cement setting is an exothermic process, i.e., one during which heat is emitted, modification of the binder composition has a significant effect on changes in the initial period of its hydration. Therefore, it is possible to relate the rate and intensity of chemical reactions occurring in the initial hours of the cement matrix formation process to the amount of heat released during this period. The extremes occurring on the hydration heat curve as a function of the material maturation time are usually indicative of significant changes taking place in the early age structure of the material being formed.

Therefore, in our study, it was attempted to determine the effect of the newly applied NA on the rheological test results of the FA-modified cement pastes. The results obtained were then correlated with the changes observed in the structure of the composites between 4th and 24th hour of their curing.

## 3. Materials

### 3.1. Properties of Materials Used to Manufacture the Cement Pastes

In order to determine the NA effect on the cement binder with FA, cement paste samples were made. Two types of pastes were used in this study, each containing 20% FA as a partial replacement for the cementitious binder. The pastes were differentiated by the NA content. The reference mix (20FA0NA) did not contain the NA. The second composite, on the other hand, contained 4% of the NA (20FA4NA). A ready-made aqueous solution with nanocrystals of hydrated calcium silicates—the Basf Master X-Seed 100 admixture—was used for this purpose. No additional plasticizers were necessary during the preparation of the samples. The amount of individual components in the cement paste mixtures is shown in Table 1.

The OPC CEM I with a 28-day strength of 32.5 MPa was used to prepare both pastes. The chemical composition of the cement used is shown in Table 2.

In contrast, Table 3, Table 4 and Table 5 show the chemical composition of the FA used (Table 3), its important physical parameters (Table 4), and the percentage composition of the different particle size fractions of the waste used (Table 5).

In addition, Table 6 summarizes the important physical properties of the NA.

### 3.2. Properties of Materials Used to Make Cement Pastes

The specimens for compressive strength and for the shrinkage testing were made according to the standard recommendations [75]. The samples were made in tripartite molds with each chamber having internal dimensions of 160 × 40 × 40 mm. The molds were lubricated inside with a layer of a non-reactive antiadhesive agent, which prevents the mixture from sticking during setting and makes it easier to unmold the specimens. They were then filled with the mixture a few mm above half height and the first vibration was performed. After thorough vibration, the molds were filled in their entire volume. Excess material was removed with a steel trowel. Then, the second vibration was carried out. Both vibration cycles were carried out on the SWE-08 vibrating table which has a vibration frequency of 52.7 Hz and the vibration amplitude is 0.67 mm. The vibration of each layer lasted about 30 s. The specimens prepared in this way were left to set in the molds under laboratory conditions—temperature 20 °C ± 5 °C, protected from water loss. In the next stage, the samples were demolded and marked. The samples were unmolded immediately before testing.

## 4. Methods

The main objective of the conducted studies was to evaluate whether it is possible to reduce the negative effect of delaying very early curing processes in the FA-containing cementitious composites, as a result of the application of modern NA with active C-S-H phase seeds. Therefore, most of the presented studies were carried out within a few or several hours after the pastes were made. The mechanical parameters, heat of hydration and microstructural tests were performed on the first day of pastes curing after: 4, 8, 12 and 24 h. Only the shrinkage test, which due to its nature manifests itself in its full range only after a longer period of time, was performed between 7 and 90 days after the preparation of the samples. Details of the performance of each test are described in the following subsections.

### 4.1. Tensile Strength under Bending and Compression

The flexural tensile strength test *f_cf_* was carried out according to [75]. Three specimens were prepared for each series and test period. The specimens during the test were positioned so that the molding surface was perpendicular to the applied load. The tensile strength of the pastes was carried out by bending the beams. After testing, the specimens were used to perform the compressive strength test. From each series, six specimens were obtained for the compressive strength test. The testing was conducted on an Advantest 9 press from Controls using a special apparatus for the flexural strength testing. The strength of each specimen was determined using the equation according to [75].

The compressive strength test *f_c_* was performed also according to [75]. The experiments were carried out in a CONTROLS Advantest 9 press using an arm with a maximum load of 250 kN and the compression testing equipment. Halved specimens from the tensile strength test were inserted into the test fixture and then compression was performed on their 40 × 40 mm faces. The specimens were compressed in the direction perpendicular to the paste molding. The compressive strength was determined according to [75]:

### 4.2. Heat of Hydration

The study was performed in a non-adiabatic non-isothermal differential microcalorimeter BMR. A 5 g sample was placed in the measuring cylinder. After stabilizing the parameters of the apparatus, water was introduced into the cylinders so that the appropriate water/binder ratio of 0.3 was maintained. The total heat effect Q(t) was determined according to [58]

### 4.3. Microstructural Investigations

The microstructural analyses of the composites in question were performed by a scanning electron microscope (SEM) on samples of prepared cement pastes. The test specimens had rectangular shapes and approximate dimensions of 10 × 10 × 3 mm.

Microstructural investigations were carried out using the SEM—QUANTA FEG 250. For each of the composites the images were taken at the same magnifications, i.e., 8000 and 16,000 times and the same reference scales, i.e., 10, and 5 µm. For each type of material and each time period the images were taken on 6 samples. 30 images were taken for each sample (a total of 120 images for each paste series), from which representative images were selected. On the SEM images, the following were marked or described: areas with the FA grains, areas with clearly distinguishable phases, e.g., C-S-H, microstructure of contacts between FA and cement matrix, e.g., view of defective or dense interfaces.

The primary objective of the microstructural study was to identify changes in the structure of the cement matrix containing the CSH seeds. The results were then related to the changes in the amount of heat released—during the paste maturation in different time periods—which were observed on the graphs made as a result of the hydration heat tests.

### 4.4. Rheological Shrinkage Test

Samples for the rheological shrinkage testing were prepared according to the recommendations of standard [74]. Samples of 160 × 40 × 40 were made in tripartite molds. Three samples for each series were prepared for testing. While forming the beams, steel tenons were mounted on their smallest side surfaces, centrally. After filling the molds, they were stored for the first 24 h in a climatic chamber; temp = 20 °C, RH = 90%. After 24 h, the samples were demolded and the first measurement was made. During the whole research cycle the samples were kept in a desiccator.

The tests were conducted in a Graf–Kaufman apparatus previously calibrated on the reference sample. Measurements of average strain $\varepsilon_{m}$ were conducted initially at 24-h intervals and then at 3-day intervals for 90 days.

The $\varepsilon_{m}$ of the samples was determined from the equation that took into account: the result of the first measurement, measurement result after time and measured length of the beam. The mean value of the measurements of all 3 samples for a given series was taken as the result of the $\varepsilon_{m}$ test.

## 5. Result and Discussion

### 5.1. Compressive Strength and Tensile Strength at Bending

The results of flexural and compressive tensile strength along with standard deviations (δ) are summarized in Table 7 and Table 8, respectively.

Analyzing the results obtained, it should be noted that the samples of both series tested after 4 h had strength values equal to 0 MPa due to the fact that it was not possible to demold them without destroying or damaging them under their own weight.

The first tests were possible only after 8 h of curing (Table 7 and Table 8). The NA series also showed a significant increase in strength compared to the reference paste. After 8 h of testing, the compressive strength was nearly 4 times higher for the NA-modified sample. In contrast, the tensile strength during this period was more than 2 times higher for the 20FA4NA. After 12 h, the NA-modified samples had almost 3 times higher *f_cm_* and still significantly higher *f_ctm_*. However, during 12 consecutive hours of pastes maturation, these differences began to blur. After 24 h the influence of NA was still significant, but it was less than 2-fold. Therefore, in order to accurately diagnose the dependence and analyze the rheological processes taking place in the samples, further experiments were carried out, i.e., the hydration heat and microstructural analyses using SEM.

### 5.2. Heat of Hydration

**Figure 1:**
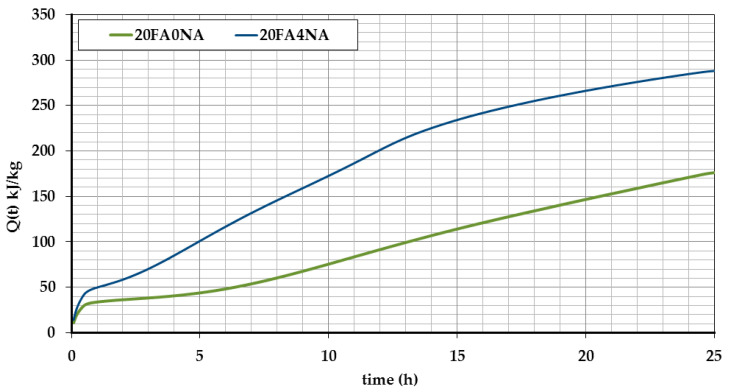
The accumulated amount of heat released over time.

**Figure 2:**
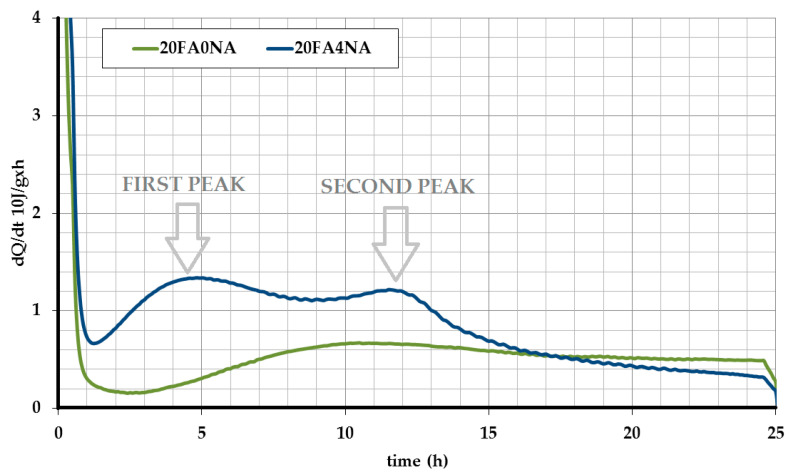
The amount of heat released over time.

The results of the hydration heat for the analyzed pastes during the first 24 h of their curing are shown in Figure 1 and Figure 2. Figure 1 shows the heat release in time, while Figure 2 shows the total heat release during the test.

The amount of heat released over time depends on the reactions occurring during the setting of the cement matrix. The main changes that take place are the hydration of the clinker phases. The hydration and pozzolanic reactions increase the heat release over time. These processes can be further catalyzed by the presence of, e.g., chemical admixtures in the binder composition.

The greatest amount of heat is released during the hydration of the calcium trialuminate (C3A). This reaction, however, is slowed down by the addition of setting time regulators (e.g., gypsum) to the cement and the conversion of C3A to the ettringite. Thus, the C3S hydration and somewhat later C2S mainly occurs. Hydration of alite as well as belite is mainly responsible for the setting of the cement matrix as well as its early mechanical strength.

**Figure 3:**
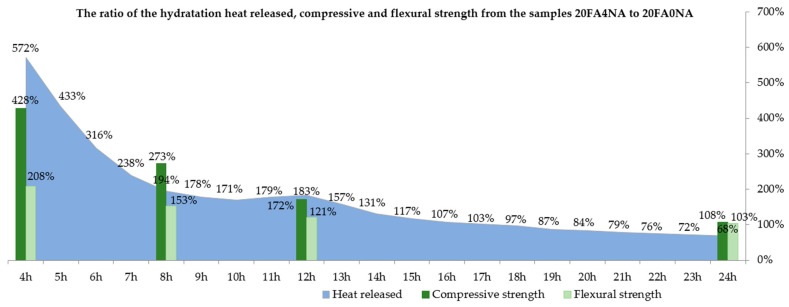
The percentage increase in the amount of heat released as well as *f_cm_* and *f_ctm_* over time.

Analyzing the graphs shown in Figure 1 and Figure 2, one should notice a significant increase in the amount of hydration heat released, observed after the NA matrix modification. Figure 3 compares the values of the total amount of heat released at different time periods for both analyzed composites. This figure also shows the percentage increases in both strength parameters after the application of active NA.

When analyzing the obtained results, it is clearly visible that the NA, after 4 h, caused an increase in the amount of heat released almost 5 times. This means that both in the space of the cement grains and in the voids (which was helped by the NA), a reaction took place and the formation of crystalline phases, mainly silicate gel. The use of NA allowed for a more effective use of both the cement itself and, additionally, the use of free spaces between the grains to create a compact and durable structure. A much greater amount of heat released in the NA-modified samples is visible during the first 16 h of the mixture curing. The strength increase in this period is also confirmed by the previously conducted strength tests of the pastes (Table 7 and Table 8). This tendency is clearly visible in Figure 3.

The graphs of heat released over time, in the case of NA modified samples, apart from changes in values, also have changes in the characteristic curve for the binding. According to the commonly known models of setting and hardening of the cement matrix, with the beginning of the setting time, a rapid amount of heat released occurs, then after reaching the peak value, after several hours of testing (usually 6–10 h), the amount of heat released decreases and the end already has a decreasing tendency. This course of the heat release curve corresponds to the reference paste (Figure 2). It shows that after reaching the peak value on the curve, after about 10 h, the direction of the hydration heat curve had a decreasing tendency. On the other hand, the use of NA caused that the course of the released hydration heat was atypical. In the NA modified samples, there was a rapid increase in the amount of released heat with the time of the onset of bonding. This value after nearly 4–5 h for the 20FA4NA series began to drop sharply. However, after another 7 h, there was another increase in the amount of released heat (rehydration), which usually does not occur in the case of binding of the traditional cement matrices. This phenomenon, atypical from the point of view of the formation of the cement matrix structure, was attempted to be explained by carefully analyzing the development of structure in individual pastes during the first 24 h of their maturation. The results of these experiments will be presented and discussed in the next subsection.

In addition, as a result of the analyzes, it was also found that after 16 h of testing, the amount of heat released was lower in the NA-modified samples (Figure 2). In connection with the study of the cement matrix shrinkage, it can be concluded that the earlier intensification of exothermic reactions in this composite may have a positive effect on the matrix structure due to the postponement of the unfavorable shrinkage phenomenon to later periods and its reduction compared to the reference composite. The results of these tests will be presented in Section 5.4.

### 5.3. Mictrostructural Investigation

**Figure 4:**
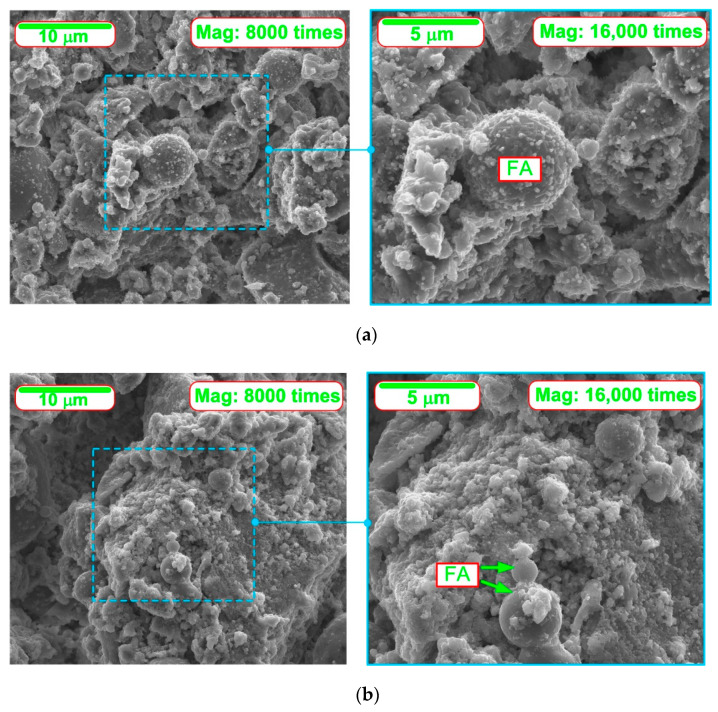

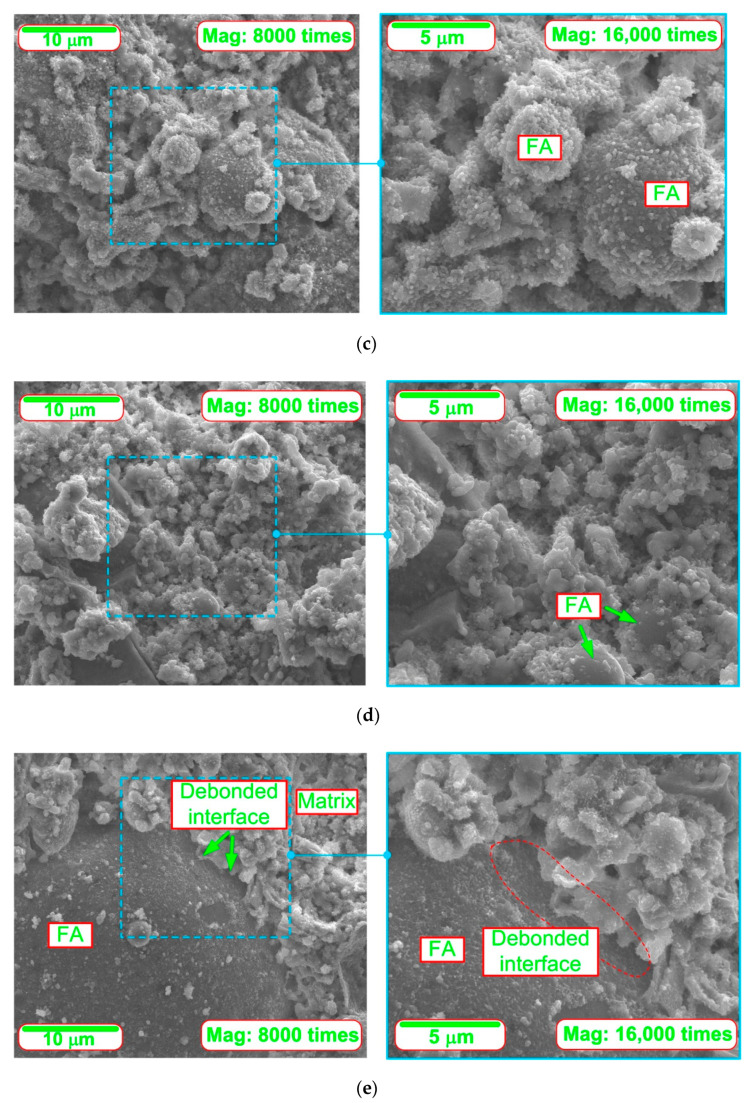

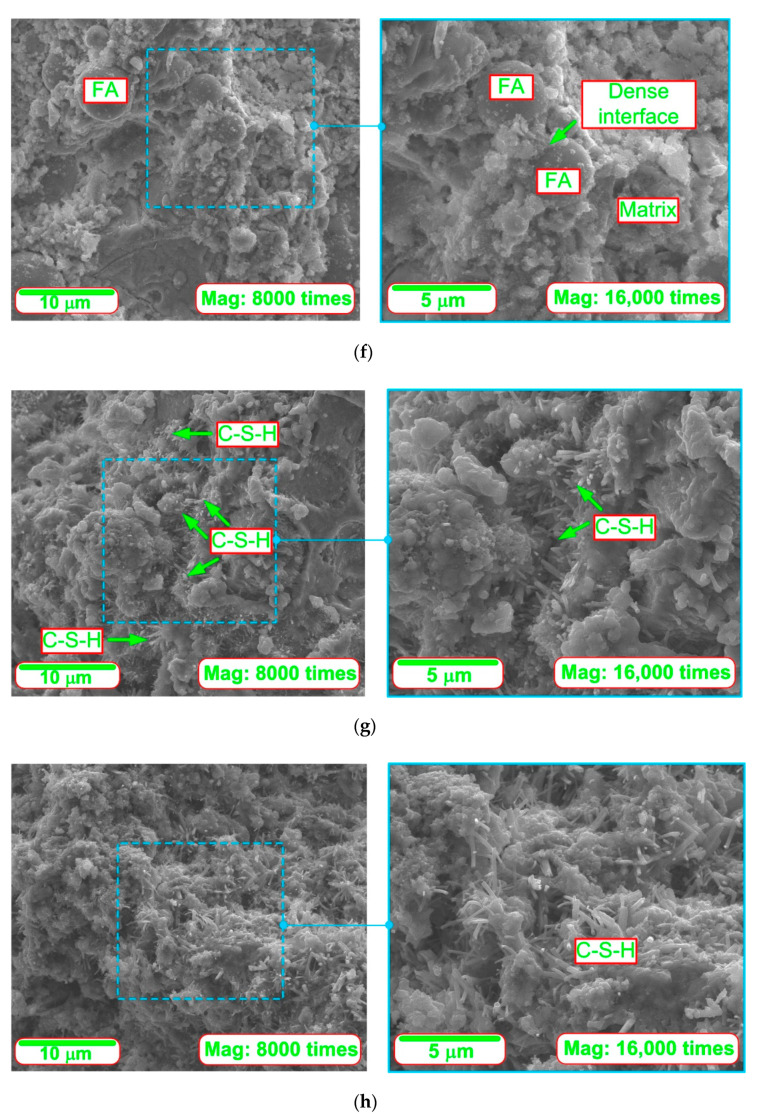
SEM micrographs of analyzed composites including FA in particular periods of curing: (**a**) 20FA0NA—4 h of curing, (**b**) 20FA4NA—4 h of curing, (**c**) 20FA0NA—8 h of curing, (**d**) 20FA4NA—8 h of curing, (**e**) 20FA0NA—12 h of curing, (**f**) 20FA4NA—12 h of curing, (**g**) 20FA0NA—24 h of curing and (**h**) 20FA4NA—24 h of curing.

Figure 4 shows sample representative SEM images of the microstructures of all the composites analyzed, after: 4, 8, 12 and 24 h of curing.

From the inspection of the SEM images at successive time periods can be observed.

A clear influence of the CSH seeds is observed, which caused a faster and more dynamic development of the cement matrix structure at a very early age. The positive effect of the modification is noticeable already in the first measurement period, in which the SEM images show significant areas of the CSH phase in the initial period of its growth (Figure 4b). In the case of the reference composite, its structure is much poorer and does not contain such a significant amount of CSH phase in a form of needles (Figure 4a). This phenomenon can be explained by the fact that during this period significant amount of the released hydration heat was observed in the case of the FA204N series (first peak marked on Figure 2), which could not be noticed in the case of the second analyzed paste (Figure 2 and Figure 3).

In subsequent time periods there was a rapid growth of both fibrous and gel-like CSH phase (Figure 4h). The structure of the composites became more and more compact with each successive period. A higher saturation of CSH phase products was also evident, which intensely filled the porous structure of the matrix during the last period of the study (Figure 4h).

Moreover, faster pozzolanic reaction of FA particles could be observed. The FA grains in concretes with NA were already strongly bonded to the matrix structure from the initial curing periods. As the hydration process progressed, their reactions in the paste were rapid, which caused the FA grains to have compact contacts with the matrix (Figure 4f). This phenomenon could be related to the time of the second distinct peak on the plot of the hydration heat of the NA-modified paste, i.e., after 12 h (second peak on Figure 2) of curing. The reference paste was characterized by a much worse structure in the ITZ of the FA grains with the matrix (Figure 4e). In this case, much less heat released during the hydration process was also observed (Figure 2). In addition, due to the catalysis of the pozzolanic reaction processes, over time the FA grains in the 20FA4NA series composite ceased to be completely visible.

### 5.4. Rheological Shrinkage

**Figure 5:**
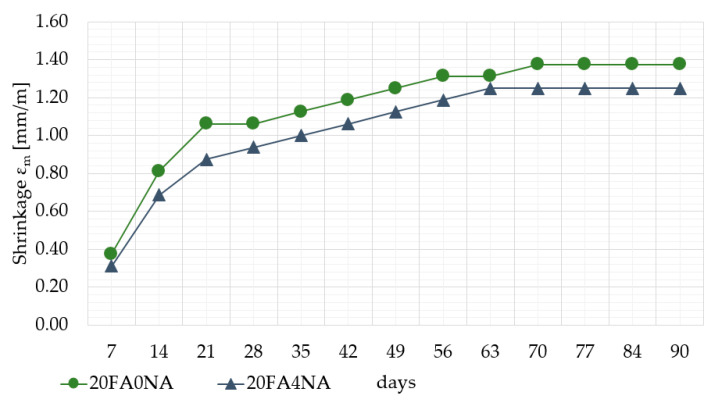
Deformations $\varepsilon_{m}$ of the tested pastes.

The study of shrinkage was carried out for 90 days from the moment of forming the samples. The results of the study in the 7-day cycle are presented in Figure 5. It is important for the early development of the matrix structure that it was not until day 7 that significant changes in contraction were found. Due to the very turbulent process of the hydration heat release in the first 24 h of the NA-modified mixtures (Figure 2) and their rapid increase in strength, it was decided to determine the impact of these processes on shrinkage.

Significant changes in measurements of the rheological shrinkage values started to be observed from the 7th day of mixtures maturation. Earlier shrinkage was almost unnoticeable and was within the limits of measurement error (in this period minimal shrinkage values of about 0.1 mm/m were recorded).

A significant increase in the rheological shrinkage was observed from the 7th day of maturation, mainly due to the drying of the pastes. Higher shrinkage was observed for the samples without NA and it was 0.25 mm/m. A clear increase in shrinkage values for both composites tested was observed between 7 and 14 days. The shrinkage values of the NA-modified pastes were lower than those in the reference pastes during the whole observation period. It can be concluded from the study that the application of NA to the paste with FA addition (Figure 5):

- Significantly reduces the shrinkage during the first 3 weeks of the curing process;
- Significantly reduces the shrinkage of this material compared to the results obtained for the traditional paste during the first 3 months after its forming.

When considering rheological shrinkage studies over 90 days, it was also observed that the NA application significantly reduces the relative shrinkage of the cement composite. After 90 days, the application of NA reduced the shrinkage value relative to the 20FA0NA samples by almost 10% (Figure 5).

## 6. Conclusions

On the basis of the conducted research on mechanical and rheological parameters as well as evaluation of the microstructure of cement pastes modified with a combined addition of FA and modern NA containing active seeds of the C-S-H phase, it can be stated that:

1. It is possible to significantly improve the early strength parameters of FA-modified cementitious composites. The use of NA in the FA-modified pastes allows to increase their strength parameters by almost 4 times after 8 h.
2. The use of NA causes a significant increase in the amount of hydration heat in the first hours of the mixture curing. It is only after 16 h that the amount of heat released over time is lower than in the reference samples.
3. After about 4 and 12 h a clear increase in the heat release can be observed in NA samples. These stages are correlated with a clear phase development in the structure of these composites, which is clearly visible in SEM images.
4. A clear relationship in the increase of the heat of hydration with the improvement of the strength parameters of composite in individual research periods after the application of the active NA with the seeds of CSH phase was also observed.
5. Despite the higher amount of the heat release in the NA paste, there was no negative effect of the modification on the shrinkage of this composite at its later stage. Moreover, the reduction in the amount of the heat release during the further curing period of the modified cementitious matrix may have contributed to a reduction in the shrinkage as well. The NA application had a positive effect on the shrinkage value in the whole analyzed measurement range, i.e., up to 90 days of material curing.
6. On the basis of SEM investigations, a clear effect of C-S-H phase seeds was observed, which not only caused a successive and dynamic development of the cement matrix structure, but also accelerated the FA pozzolanic reactions.
7. The proposed NA in the form of active CSH seeds is a modern and innovative form of accelerating the curing of concretes at a very early age, leading to the formation of a more homogenized and compact cement matrix structure in concretes containing FA.

## Figures and Tables

**Table 1:** Mixture proportions.

Component	20FA0NA	20FA4NA
OPC CEM I 32.5R, Ożarów	1.60	1.60
FA, Puławy Combined Heat and Power Plant	0.40	0.40
NA CSH—Master X-SEED 100	-	0.08
Water	0.60	0.52

**Table 2:** Chemical composition of the OPC.

Constituent	SiO_2_	Al_2_O_3_	Fe_2_O_3_	CaO	K_2_O	SO_3_	MgO	Cl
(wt.%)	15.00	2.78	2.72	71.06	1.21	4.56	1.38	0.08

**Table 3:** Chemical composition of the FA.

Constituent	SiO_2_	Al_2_O_3_	Fe_2_O_3_	K_2_O	SO_3_	MgO	CaO	P_2_O_5_	Ag_2_O	BaO	TiO_2_	SrO	LOI *	Σ:
(wt.%)	55.27	26.72	6.66	3.01	0.47	0.81	2.35	1.92	0.1	0.1	1.89	0.22	4.66	99.52

SiO_2_ + Al_2_O_3_ + Fe_2_O_3_ = 88.65% ≥ 70.00%; * LOI—Loss of ignition.

**Table 4:** Physical parameters of the FA.

Parameter	Average Value
Bulk density (g/cm^3^)	2.14
Specific surface area (cm^2^/g)	2944
Fineness (%)	39.2

**Table 5:** Division of the FA into fractions.

Particle Size (μm)	Volume (%)
0.01–2	1.12
2–20	23.72
20–50	20
50–100	21.59
100–250	25.49
250–500	5.99
500–1000	1.09
1000–2000	0.93

**Table 6:** Physical parameters of the NA.

Parameter	Average Value
Density of suspension (g/cm^3^)	1.135 ± 0.02
pH	11.5 ± 2
Chloride content (%)	<0.1
Alkali content (%)	<4.0

**Table 7:** Compressive strength test results—*f*_cm_.

Series Designation	*f*_cm_ (MPa)	±δ (MPa)
20FA0NA-4 h	0.00	0.00
20FA4NA-4 h	0.00	0.00
20FA0NA-8 h	1.19	0.07
20FA4NA-8 h	5.11	0.17
20FA0NA-12 h	3.32	0.12
20FA4NA-12 h	9.06	0.45
20FA0NA-24 h	18.87	0.73
20FA4NA-24 h	32.41	0.82

**Table 8:** Tensile strength test result—*f*_ctm_.

Series Designation	*f*_ctm_ (MPa)	±δ (MPa)
20FA0NA-4 h	0.00	0.00
20FA4NA-4 h	0.00	0.00
20FA0NA-8 h	0.04	0.00
20FA4NA-8 h	0.09	0.02
20FA0NA-12 h	0.07	0.02
20FA4NA-12 h	0.11	0.03
20FA0NA-24 h	0.38	0.02
20FA4NA-24 h	0.46	0.04

## Data Availability

No new data were created or analyzed in this study. Data sharing is not applicable to this article.
